# Histone H1 Subtypes Differentially Modulate Chromatin Condensation without Preventing ATP-Dependent Remodeling by SWI/SNF or NURF

**DOI:** 10.1371/journal.pone.0007243

**Published:** 2009-10-01

**Authors:** Jaime Clausell, Nicole Happel, Tracy K. Hale, Detlef Doenecke, Miguel Beato

**Affiliations:** 1 Centre de Regulació Genòmica (CRG), Universitat Pompeu Fabra, Barcelona, Spain; 2 Department of Molecular Biology, Institute for Biochemistry and Molecular Cell Biology, University of Goettingen, Goettingen, Germany; 3 Department of Paediatrics, Christchurch School of Medicine, University of Otago, Christchurch, New Zealand; Texas A&M University, United States of America

## Abstract

Although ubiquitously present in chromatin, the function of the linker histone subtypes is partly unknown and contradictory studies on their properties have been published. To explore whether the various H1 subtypes have a differential role in the organization and dynamics of chromatin we have incorporated all of the somatic human H1 subtypes into minichromosomes and compared their influence on nucleosome spacing, chromatin compaction and ATP-dependent remodeling. H1 subtypes exhibit different affinities for chromatin and different abilities to promote chromatin condensation, as studied with the Atomic Force Microscope. According to this criterion, H1 subtypes can be classified as weak condensers (H1.1 and H1.2), intermediate condensers (H1.3) and strong condensers (H1.0, H1.4, H1.5 and H1x). The variable C-terminal domain is required for nucleosome spacing by H1.4 and is likely responsible for the chromatin condensation properties of the various subtypes, as shown using chimeras between H1.4 and H1.2. In contrast to previous reports with isolated nucleosomes or linear nucleosomal arrays, linker histones at a ratio of one per nucleosome do not preclude remodeling of minichromosomes by yeast SWI/SNF or *Drosophila* NURF. We hypothesize that the linker histone subtypes are differential organizers of chromatin, rather than general repressors.

## Introduction

The expansion in length of genomes and the increase in organism complexity during evolution were made possible by the emergence of the eukaryotic cell with an organelle, the nucleus, specialized in compacting, storing, manipulating and replicating DNA. The first level of packaging and compacting of DNA is achieved by wrapping the double helix around a cylinder of basic proteins, the four core histones H3, H4, H2A and H2B, which neutralize the negative charges of the DNA phosphodiester chain. Core histones exhibit a characteristic histone fold domain and are organized as a symmetric octamer with a central H3/H4 tetramer flanked by two H2A/H2B dimers. This structure exposes a left-handed superhelical ramp of positively charged amino acid residues along which 147 DNA bp wrap forming nucleosome core particles (NCP) [1]. NCP are connected by linker DNA that is in contact with and organized by another family of basic proteins known as the linker histones. The binding of a linker histone molecule to the core particle and linker DNA leads to the formation of a new structure, the nucleosome with a variable length of linker DNA [2]. In comparison with the core histones, histone H1 lacks the histone fold domain and belongs to the winged helix family of DNA-binding proteins. Histone H1 is located at the dyad axis of the nucleosome, in contact with the entry and exit sites of the nucleosomal DNA, and is critical in organizing [3] and stabilizing maximal nucleosome compaction within the chromatin fibre [4], [5]. In spite of this crucial role, little is known about the physiological function of histone H1, in part due to the large diversity of subtypes.

Eleven different H1 subtypes have been identified in mammals [6], [7]. Seven are somatic (H1.1–H1.5, H1.0 and H1x, with the nomenclature proposed by Doenecke and coworkers [8]), three are spermatogenic (H1t, H1T2 and HILS1) and one oocyte specific (H1foo) [9]–[13]. They differ in timing of expression [14], extent of phosphorylation [15], turnover rate [16]–[18], chromatin binding affinity [19], [20], and evolutionary stability [21]. Differences in DNA condensing capacity [19], [22], [23] and in their preference for euchromatin or heterochromatin [18] have also been demonstrated for some subtypes. The ‘replacement subtype’ H1.0 was initially described in highly differentiated, non-dividing cells [9] and expression of its gene can be turned on by inducers of differentiation (reviewed by Zlatanova and Doenecke [24]). The H1.1 subtype seems to be restricted to thymus, testis, spleen, lymphocytic and neuronal cells [25], [26]. Expression of the testis-specific H1t [27] was found to be restricted to pachytene spermatocytes during the meiotic prophase [28]. However, recent mass spectrometry data indicate that H1t is also present in spleen and in lymphocytes [29].

H1 is essential for murine development. While mice lacking one or two of the somatic H1 genes develop normally [30], when three H1 genes (H1.2, H1.3 and H1.4), were inactivated, mice die by mid-gestation with a broad spectrum of defects [31]. Immunodepletion of H1 was shown to produce aberrant mitotic chromosomes that could not segregate properly [32]. Depletion of a single subtype, H1.2, produced G1 arrest in T47D and MCF10A breast epithelial cell lines and apoptosis in MCF7 cells, another breast cancer derived cell line [33]. Moreover, depletion of H1.4 caused cell death in T47D cells, providing the first report of a crucial role for a H1 subtype in the survival of a human cell type. Expression of a different subset of genes was altered in each of these H1 knock-downs, again suggesting differential functions for the various H1 subtypes in somatic cells [33].

Several studies have attempted to determine the binding affinity of H1 to chromatin. Orrego and co-workers [20] used H1 subtypes from rat brain to determine their relative affinities for nucleosomal arrays, classifying them into groups of high affinity (H1.3, H1.4 and H1.0), intermediate affinity (H1.5 and H1.2) and low affinity (H1.1). On the other hand, Talasz [19] used purified H1 subtypes from mouse liver to measure their binding to mononucleosomes, classifying them as high (H1.2, H1.3 and H1.4), intermediate (H1.1) and low (H1.5) binding affinity. These *in vitro* results differ from those obtained in a cell-based study where GFP protein was fused to the human H1 subtypes to determine their turnover using Florescence Recovery After Photobleaching (FRAP) [18]. This study showed that there are tight binding (H1.4 and H1.5), intermediary binding (H1.3 and H1.0) and weaker binding H1 subtypes (H1.1 and H1.2).

Reports on the effect of linker histones on the level of chromatin compaction are incomplete and partly contradictory. According to Liao and Cole [22], H1.2 weakly aggregates dinucleosomes in comparison to H1.3, H1.4 and H1.5. Khadake and Rao [23] showed that H1t and H1.1, are less condensing than H1.2, H1.3, H1.4 and H1.5. However, Talasz *et al.* [19] determined H1.1 to be the strongest condenser while H1.5 was the subtype that aggregated polynucleosomes the least followed by H1t and H1.2.

Linker histones inhibit the spontaneous and thermally induced sliding of histone octamers [34], [35]. Due to this stabilizing property, they were postulated to inhibit chromatin remodeling as shown for mononucleosomes [36], dinucleosomes [37] or nucleosomal arrays [38]. However, other studies have shown that chromatin remodeling is possible in the presence of histone H1, using either mononucleosomes [39], minichromosomes [40] or *in vitro* reconstituted chromatin fibres [41]. The differences with the former studies could be due to the remodeling complexes, the H1 stoichiometry, or the *in vitro* conditions used.

This study attempts to unravel the contradictions with regards to the properties of the somatic histone H1 subtypes by using native-like chromatin, namely minichromosomes assembled with pre-blastodermic *Drosophila melanogaster* embryo extracts. We have used two sets of human H1 subtypes expressed in bacteria and in yeast to investigate their affinity for chromatin, and their effect on nucleosome spacing and chromatin compaction. Our results eliminate most of the contradictions between previous *in vitro* and cell based studies and allow us to classify the somatic H1 subtypes into three categories based on their chromatin compacting properties. Moreover, we have not detected significant differences in the ability of two different ATP-dependent remodeling complexes to remodel minichromosomes deprived of histone H1 or containing each of the histone H1 subtypes.

## Results

### Influence of H1 subtypes on nucleosome spacing and their affinity for chromatin

**Figure 1:**
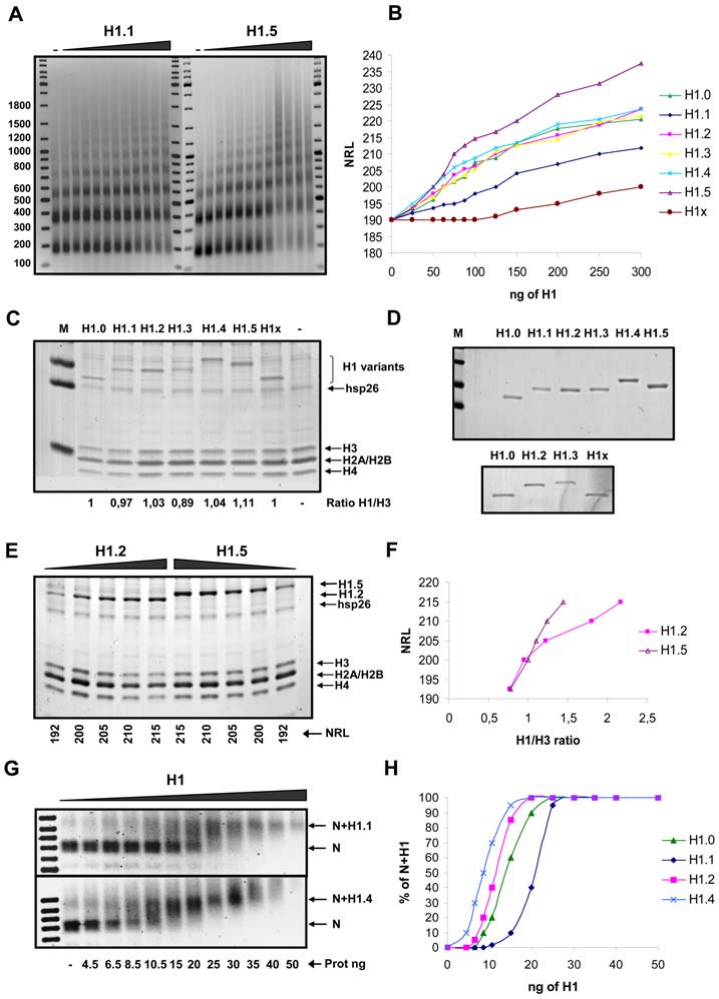
Affinity of histone H1 subtypes for chromatin. (A) Nucleosome ladders obtained after micrococcal nuclease (MNase) digestion of minichromosomes assembled in preblastodermic *Drosophila* embryo extracts (DREX) with increasing amounts of histone H1 subtypes. Results with H1.1 and H1.5 are shown as an example. DNA size markers are shown on both sides of the MNase digested samples. Numbers on the left indicate fragment length in base pairs. (B) Graphic representation of the Nucleosomal Repeat Length (NRL) calculated from experiments similar to that shown in (A) with the amount of each H1 subtype indicated in nanograms. (C) Minichromosomes, assembled with each H1 subtype to yield a NRL of 200 bp, were precipitated with buffer containing 20 mM MgCl_2_, and their proteins were electrophoresed on a 16% SDS-Polyacrylamide gel (SDS-PAGE), and visualized by Coomassie G-250 staining. The bands were quantified using Quantity One software (Bio-Rad) and the ratio between each H1 and H3 is shown below the corresponding gel lane. H1.4 runs slower than the other H1 subtypes as it contains a FLAG tag. Abbreviation and Symbol: M, Marker; -, without H1. (D) Staining of 2 µg of each purified H1 subtypes (H1.0 to H1.5) with Coomassie G-250. The lower panel is from a separate experiment that included H1x and a subset of the other subtypes for comparison. (E) Minichromosomes assembled with increasing amounts of H1.2 and H1.5 corresponding to the NRL indicated below, were purified as in (C). Proteins were separated by electrophoresis and stained with Coomassie G-250. (F) The intensity of the bands corresponding to H3, H1.2 and H1.5 in (E) were quantitated, normalised according to their different staining ability shown in (D), and the NRL plotted against the H1/H3 ratio. (G) Mononucleosomes assembled by salt dialysis on 100 ng of a 220 bp DNA fragment were incubated for 20 minutes with increasing amounts of H1.1 and H1.4 and analysed on a 0.7% agarose gel. The position of the mononucleosome without H1 (N) and with H1 is indicated on the right margin. (H) Graphic representation of the extent of H1.0, H1.1, H1.2 and H1.4 binding, calculated from the band shift experiments as shown in (G).

We first characterised the nucleosome repeat length (NRL) of chromatin assembled in preblastodermic *Drosophila melanogaster* embryo extracts (DREX) supplemented with the linker histone subtypes. Since these extracts do not contain histone H1 [42], the effect of adding histone H1 can be easily measured [43]. Under the conditions of the assay in the absence of histone H1 the NRL was 190 bp. With increasing amounts of H1 subtypes expressed in *E. coli* the NRL increased almost linearly up to a point where the slope of the curves diminished (Figures 1A and 1B). The initial slopes of these titration curves were different for the various H1 subtypes, although an equal amount of each subtype was used. At the lowest protein concentration, H1.5 increased NRL most efficiently followed by H1.4, which had lower spacing activity (Figure 1B), but when expressed in yeast behaved like H1.5 (data not shown). The subtypes H1.2, H1.3 and H1.0 were indistinguishable in this assay, while higher protein concentrations of H1.1 were necessary to increase the NRL. To reach a NRL of 200 bp, twice the concentration of H1.1 was needed compared to H1.0 (and H1.2 and H1.3). H1x was the somatic subtype that required the highest concentration to reach a certain NRL (Figure 1B).

The results shown above could reflect differences in the affinity of the H1 subtypes for chromatin or differences in their nucleosome spacing ability. To distinguish between these two possibilities, we measured the amount of each histone H1 bound to chromatin at a NRL of 200 bp. We chose this spacing because it is within the linear range of increasing NRL as a function of the H1 concentration (Figure 1B) [44]. To selectively precipitate the assembled minichromosomes from the reconstitution reaction we adapted a method previously used to purify short chromatin fibres [41] based on their precipitation by MgCl_2_ [45], an effect that can be modulated by monovalent cations [46]. A higher concentration of MgCl_2_ was needed to precipitate minichromosomes without H1 than with each H1 subtype, with the exception of H1.1 (Figure S1). Finally, minichromosomes containing each of the H1 subtypes were precipitated and their histone content analysed in a polyacrylamide gel (Figure 1C). The bands corresponding to each H1 subtype and histone H3 were quantitated by densitometry (Quantity One, Bio-Rad) and the ratio between them calculated. With all H1 subtypes the H1/H3 ratio was close to one. The small differences are likely due to the different affinity of each subtype for Coomassie, as shown by the staining of 2 µg of each purified H1 subtype (Figure 1D). We conclude that equal amounts of each subtype were bound to minichromosomes at 200 bp of NRL. Therefore, the varying amounts of each H1 subtype needed to increase the NRL (up to 200 bp) reflect their differences in affinity for chromatin.

To correlate NRL with histone content over a wider range of concentrations we assembled and purified minichromosomes with amounts of H1.2 or H1.5 leading to NRL of 192, 200, 205, 210 and 215 bp (as in Figure 1B). The histone content incorporated into the minichromosomes was analysed by MgCl_2_ precipitation and PAGE (Figure 1E). Although a similar amount of either subtype was bound at 200 bp NRL, much higher amount of bound H1.2 was needed to obtain longer NRL (Figure 1F). Thus, above the stoichiometry of one H1 per nucleosome, these two subtypes differ markedly in their ability to space nucleosomes.

The NRL assays described above were performed with H1 expressed in *E. coli* except for H1.1 which was expressed in yeast. To confirm these results, the H1 subtypes were expressed in yeast and purified as previously reported [47]. Again, the high affinity subtypes H1.5 and H1.4 exhibited the highest affinity, followed by H1.3, H1.2 and H1.0 (data not shown), while H1.1 required higher amounts of protein. Therefore, the histone H1 subtypes expressed in yeast behave in a similarly manner to those expressed in bacteria.

We then tested whether the differences in affinity of the H1 subtypes for minichromosomes could be reproduced with isolated mononucleosomes. We assembled mononucleosomes without H1 on a 220 bp DNA fragment and using HeLa core histones, which are extensively conserved in relation with Drosophila core histones. We measured the affinity of the H1 subtypes in a band shift assay. The results (Figure 1G and quantification in Figure 1H) showed that the mononucleosome band is more efficiently retarded by H1.4, followed by H1.2, H1.0 and finally H1.1. Thus, the differences in affinity of the various subtypes for minichromosomes reflect their relative affinities for mononucleosomes. We can distinguish three subgroups: high affinity subtypes, H1.5 and H1.4; intermediate affinity subtypes, H1.2, H1.3 and H1.0; and low affinity subtypes, H1.1 and H1x.

### Histone H1 subtypes have different chromatin compacting capacity

**Figure 2:**
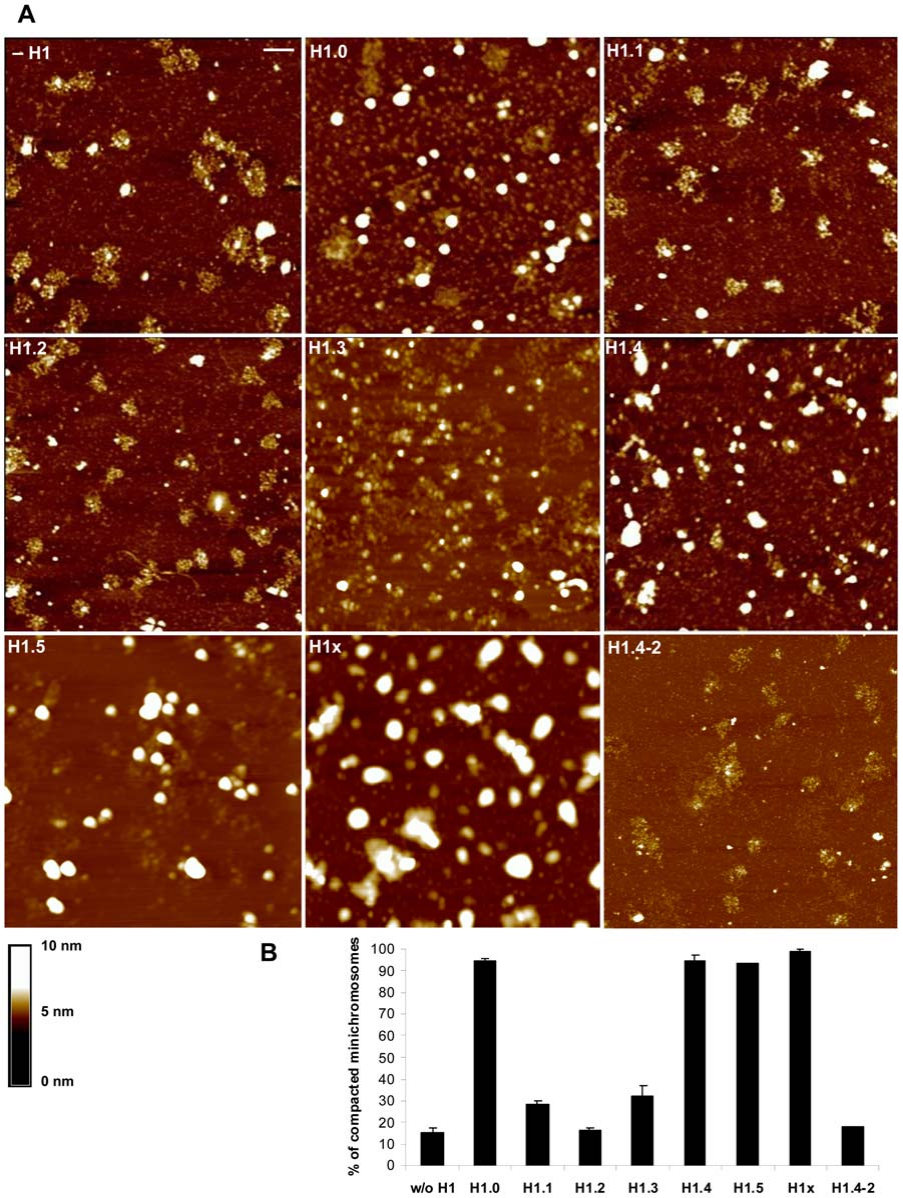
Influence of H1 subtypes and the H1.4–2 chimera on chromatin compaction. (A) Minichromosomes were assembled without H1 or with the indicated H1 subtype or the chimeric protein H1.4–2, all at concentrations yielding a NRL of 200 bp. Minichromosomes were purified through a sucrose gradient and a sucrose cushion, resuspended in 20 mM KCl containing buffer, immobilized on an APTES mica surface [48] and visualised using TMAFM (Tapping Mode Atomic Force Microscopy). The horizontal bar represents 200 nm and the vertical scale (in nm) is shown below; the grid size is 2 µm. Nucleosomes appear as balls of 11 nm and compacted minichromosomes as globules of 50–60 nm in diameter. Single nucleosomes are lost from the minichromosomes during the fixation process and appear as individual balls of approximately 11 nm. (B) The percentage of compacted minichromosomes was calculated from at least two independent experiments and is shown in the bottom diagram, with the error bars corresponding to S.E.M.

**Figure 3:**
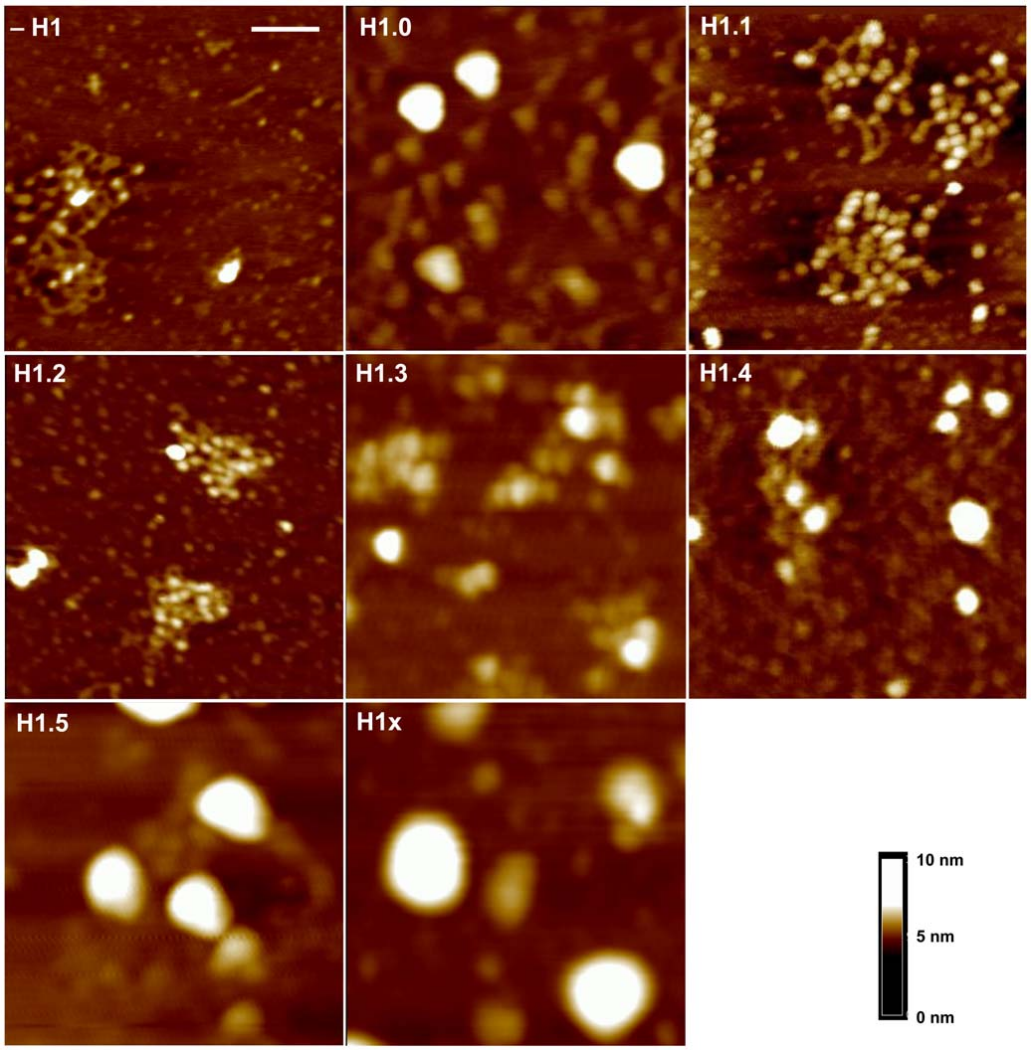
500 nm magnifications of minichromosomes assembled without or with each somatic H1. The same techniques as in Figure 2 were used. The horizontal bar corresponds to 100 nm and the grid size is 500 nm. The vertical scale is shown.

To compare the H1 subtypes in terms of their capacity to compact chromatin we used Atomic Force Microscopy (AFM). Minichromosomes containing amounts of each linker histone subtype yielding an equivalent NRL (200 bp) and comparable stoichiometry, were reconstituted using DREX. To purify the minichromosomes, the assembly reactions were ultracentrifuged through a sucrose gradient and the fractions containing minichromosomes were pooled and concentrated by centrifugation through a sucrose cushion. The pellets were resuspended in a buffer containing 20 mM KCl and deposited on an APTES-mica surface previously treated with glutaraldehyde [48]. The surface was dried and the minichromosomes visualised with a Nanoscope III AFM. Although the NRL and the linker histone content were equivalent in all samples, the minichromosomes exhibited differing degrees of compaction that depended on the H1 subtype incorporated (Figure 2). Nucleosomes were clearly recognizable as individual 11 nm particles (see the horizontal scale bar and vertical scale in Figure 2A; the 500 nm magnifications are shown in Figure 3) in minichromosomes containing H1.1 and H1.2, which showed a decondensed structure similar to that found in the absence of histone H1 (−H1). In images of minichromosomes containing H1.3, nucleosomes were still recognizable though less clearly, as they were either forming groups or too close to be distinguished by AFM. Minichromosomes with H1.0, H1.4, H1.5 or H1x, formed larger condensed structures and individual nucleosomes were not recognizable (Figure 2A). These structures resemble the globules described by Dubochet *et al.* [49] when studying SV40 minichromosomes. Finally, minichromosomes containing H1x seemed to aggregate between each other leading to the formation of larger clumps.

We measured (Figure 2B) the differences between the images obtained with the various H1 subtypes by counting the number of compacted and decompacted minichromosomes in each preparation. A minichromosome was considered compacted when individual nucleosomes were not recognizable and the diameter of the whole particle was within the limits (50–60 nm) of spheres or globules consisting of 25 nucleosomes.

Figures 2 and 3 allow us to classify the H1 subtypes according to their chromatin compacting ability into: weakly compacting H1.1 and H1.2, intermediate compacting H1.3, and highly compacting, H1.0, H1.4, H1.5 and H1x.

### The variable C-terminal domain of H1 is the main determinant of chromatin affinity, nucleosome spacing and chromatin condensation

Since the globular domain (GD) is conserved from H1.1 to H1.5, the differences in the ability of the H1 subtypes to condense chromatin must reside in their terminal tails. To test this hypothesis and to identify the relevant domain, the C-terminal tail of the highly condensing subtype H1.4 was substituted with the C-terminal tail of the weakly condensing subtype H1.2, creating a H1.4–2 chimera. As shown in Figure 2A and 2B, the chimera has a lower compacting ability than H1.4 as a result of the C-terminal tail of H1.2, demonstrating that the C-terminal domain is the main determinant of the compacting capacity.

**Figure 4:**
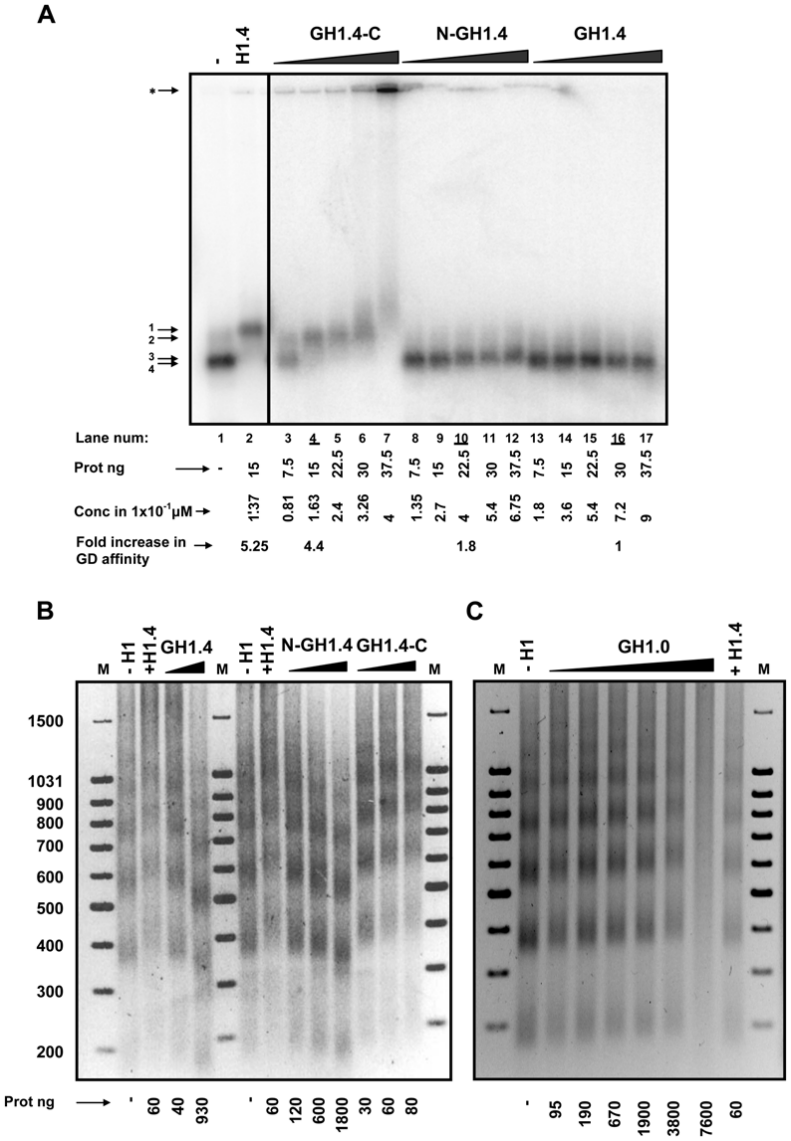
Role of H1 domains in chromatin affinity and nucleosome spacing. (A) Centrally positioned mononucleosomes were assembled on a 220 bp DNA fragment corresponding to the nucleosome B in the MMTV promoter. After a glycerol gradient purification, mononucleosome particles were incubated with increasing amounts of the following H1.4 domain mutants: the globular domain (GH1.4), the N-terminal plus the globular domain (N-GH1.4), and the globular plus the C-terminal domain (GH1.4-C), and analyzed on a 0.7% (w/v) agarose gel. A lane with wild type H1.4 is shown as control. The different particles are numbered on the left margin: 1) Mononucleosome + H1.4; 2) Mononucleosome + GH1.4-C; 3) Mononucleosome + N-GH1.4; 4) Mononucleosome + GH1.4. The symbol ‘*’ indicates the appearance of aggregates. The amount of each protein is indicated at the base of the gel (lanes 2–17). Protein concentrations are also expressed in 1×10^−1^ µM units. (B) MNase digestion of minichromosomes assembled with the indicated linker histone. The amount of linker histone added (in nanograms per 200 ng of DNA) is indicated bellow each lane. Lettering is as in (A). Numbers on the left margin refer to the size of the markers (lane M) in base pairs. (C) MNase digestions of minichromosomes with increasing amounts of GH1.0. Also shown is the digestion in the absence of linker histone (−H1) and in the presence of H1.4 (+H1.4). Markers and the amount of protein added are indicated as in (B).

To further investigate the properties of the H1 domains, we generated domain deletion mutants of the H1.4 subtype. GH1.4 is a mutant containing only the GD, N-GH1.4 contains the N-terminal and the GD, and GH1.4-C contains the GD plus the C-terminal domain of H1.4. We assessed the binding properties of the H1.4 domain mutants to mononucleosome particles assembled on a 220 bp DNA fragment using gel retardation assays (Figure 4A; for H1.4 titration see Figure S2). Titration of increasing amounts of each protein showed that wild-type H1.4 and the GH1.4-C mutant had similar affinities, as they promoted gel retardation at similar protein concentrations (lanes 2 and 4), while N-GH1.4 and GH1.4 exhibited a much lower affinity for these particles. A weak retardation is promoted by N-GH1.4, as seen in lanes 10–12. A more precise comparison based on µM concentrations is also shown in Figure 4A. The C-terminus also conferred on H1.4 its nucleosome aggregating properties, as shown in Figure 4A (lane 7). For the concentration at which H1.4 causes aggregation, no aggregation was generated by mutants lacking the C-terminal domain.

The small changes in structure and charge of mononucleosomes that occur upon binding of GH1 in the absence of the H1 tails [50] seem not sufficient to vary the electrophoretic migration rate (Figure 4A). In several previous studies the binding of either GH5 or GH1 to mononucleosomes has been assessed by gel retardation and shown to have little effect on mononucleosome migration [51], [52].

We also assembled minichromosomes in the presence of the H1.4 domain mutants and tested their affinity for chromatin and effect on nucleosome spacing. Wild-type H1.4 and GH1.4-C had similar effects on NRL, while GH1.4 and N-GH1.4 required much higher protein concentrations to generate changes in NRL (Figure 4B). Therefore, we concluded that the C-terminal domain is also responsible for increasing the spacing between nucleosomes along the DNA molecule and is the main determinant of histone H1's affinity for chromatin.

Paradoxically, when GH1.4 and N-GH1.4 were added at higher concentrations we observed a decrease in nucleosome spacing (Figure 4B), a phenomenon that could reflect self-association of these mutants (see Discussion). Because the GD of H1.0 exhibits subtle differences in amino acid sequence compared to the rest of the somatic H1 subtypes, we also purified and tested GH1.0 in this assay. Intriguingly, GH1.0 behaved differently from GH1.4 at equivalent protein concentrations, as it increased rather than decreased NRL (Figure 4C).

Finally, minichromosomes containing the H1.4 domain mutants were purified and visualised with the AFM (Figure S3). Both terminal domains appeared to contribute to chromatin condensation, although the mutants containing the C-terminal domain induced this effect at a much lower concentration.

### Chromatin remodeling of minichromosomes assembled with H1 subtypes

**Figure 5:**
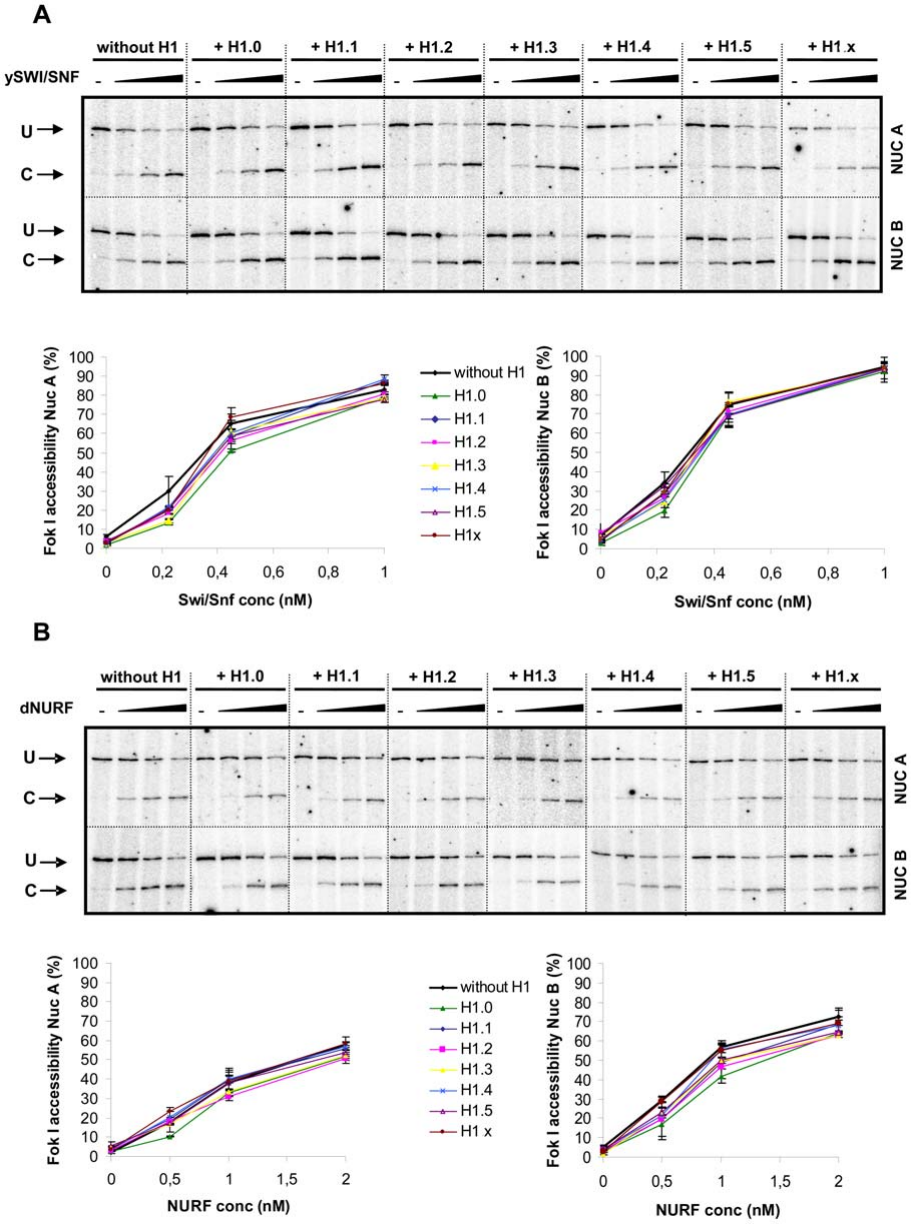
Effect of histone H1 subtypes on ATP-dependent chromatin remodeling. MMTV minichromosomes were reconstituted in the presence of the histone H1 subtypes, as indicated, purified, resuspended in a 60 mM KCl and 5 mM MgCl_2_ containing buffer, and incubated with *Fok* I that cleaves in nucleosome A and B of the MMTV promoter. Increasing nM concentrations of SWI/SNF (A) and NURF (B) complexes were added and incubated for 30 minutes at 30°C. The reaction was stopped and a linear extension PCR with a radiolabeled oligonucleotide was performed. The cleavage products were visualised in a 10% polyacrylamide denaturing gel and quantified using Image Quant software (Amersham). Minichromosomes of two independent purifications were tested and the results represented with the corresponding S.E.M in the graphs bellow. Abbreviations: Nuc A, nucleosome A; Nuc B, nucleosome B; U, uncut material; C, cut material.

We next studied the influence of the H1 subtypes on ATP-dependent chromatin remodeling. Two different ATP-dependent chromatin remodeling complexes were used, yeast SWI/SNF and *Drosophila* NURF. Yeast SWI/SNF was purified from yeast strains expressing a tagged SNF2 subunit as previously described [53] and recombinant *Drosophila* NURF was purified from baculovirus infected S9 cells by affinity chromatography via the tagged SNF2H subunit [54]. We used Fok I restriction enzyme (RE) cleavage at 30°C as a measure of the increase in chromatin accessibility generated by these chromatin remodelers. Fok I cleaves MMTV minichromosomal DNA at two sites located in separated nucleosomes over the MMTV promoter (Nuc A and Nuc B, Figure 5 and Figure S5) and therefore it gives information of the effect in two different contexts.

MMTV minichromosomes were assembled lacking H1 or containing each of the somatic H1 subtypes at concentrations yielding a stoichiometry of one H1 per nucleosome (NRL of 200 bp). The resulting minichromosomes were purified and exposed to increasing concentrations of chromatin remodelers. In the absence of remodeling activities, *Fok* I could not access its cleavage sites in minichromosomes during the 30 min incubation (Figure 5, lanes marked -). In the presence of ATP and either ySWI/SNF (Figure 5A) or dNURF (Figure 5B), the accessibility of both *Fok* I cleavage sites increased with the concentration of the remodeling complexes. None of the H1 subtypes precluded the increase in accessibility. Compared to cleavage observed in the absence of linker histones, there was no significant effect with any of the H1 subtypes despite their differing abilities to compact chromatin. We conclude that ySWI/SNF and dNURF complexes are capable of remodeling native-like chromatin purified from the *Drosophila* assembly system and containing physiological amounts of histone H1.

## Discussion

The results of this study indicate that there are marked differences in the affinity of the human somatic histone H1 subtypes for chromatin *in vitro*, similar to those found in cell based studies. Moreover somatic histone H1 subtypes differ also in their chromatin compacting properties and these differences do not simply reflect their differences in chromatin affinity. The different properties of H1 subtypes reside mainly in the long and variable C-terminal domain and do not affect the capacity of SWI/SNF and NURF complexes to efficiently remodel H1 containing minichromosomes. In the following we will discuss the possible significance of these results for the structure and function of chromatin.

### H1 subtypes affinity for chromatin

The classification of histone H1 subtypes according to the amount of protein required to generate a NRL of 200 bp yielded the following order of nucleosome spacing capacity, [H1.5, H1.4] > [H1.3, H1.2, H1.0] > H1.1>H1x. Direct measurement of the amount of linker histone bound to minichromosomes at this NRL showed that this order reflects the affinity of the H1 subtypes for chromatin. Thus, within the physiological range of about one H1 molecule per nucleosome there are no major differences in the ability of chromatin bound H1 subtypes to space nucleosomes. At higher concentrations of bound subtypes, H1.5 can generate a longer NRL than H1.2, although we have not explored other H1 subtypes at these unphysiologically high concentrations.

A similar order of affinities for the H1 subtypes was obtained for *in vitro* assembled mononucleosomes using a band shift assay. The order of affinity and consequently the capacity for binding and spacing nucleosomes was similar for each of the human H1 subtypes expressed in bacteria and in yeast. The discrepancies between our results and the order of affinities reported in some previous studies could be due to the use of H1 subtypes from different species, differences in the purification method or the chromatin interaction assay. In previous studies, H1 was purified from rat [20] or mouse tissues [19], whereas we used human recombinant proteins expressed in yeast or in bacteria. Moreover, published reports studied the affinity of H1 subtypes for preformed chromatin [20], or mononucleosomes [19], whereas we have added the H1 subtypes during the chromatin reconstitution process, thus providing an environment and a chaperone content that is closer to the physiological situation.

To our knowledge, this is the first time that the complete set of human somatic H1 subtypes have been compared in a nearly physiological chromatin environment. Our results agree with the findings obtained with GFP tagged H1 subtypes in living cells using FRAP, which identified H1.4 and H1.5 as tightly bound chromatin subtypes, H1.3 and H1.0 as intermediate chromatin affinity subtypes, and H1.1 and H1.2 as weakly bound [18]. Moreover, H1x exhibits a faster turnover rate than H1.2 [55]. This correlation with the *in vivo* studies supports the physiological relevance of our *in vitro* assays.

### H1 domains and nucleosome spacing

The mechanisms by which defined nucleosome spacing is produced, how it changes during the cell cycle and why it differs between cell types are not well understood. Different nucleosome spacings have been related to different levels of chromatin folding [56]–[58]. In general, a decrease in spacing has been associated with H1 removal and is supposed to correlate with more open chromatin states and active genes. Increasing H1 incorporation results in a gradual increase in NRL rather than in defined 20 bp steps [59]–[61]. This progressive increase in spacing could reflect the average behaviour of the nucleosomal population and we cannot exclude that individual chromatin regions change their nucleosome spacing in steps of defined length.

With domain mutants of H1.4 we show that the C-terminal domain is the major contributor to the affinity of this linker histone for chromatin. A critical role for the C-terminus of H1 in the organization of the linker region and spacing between nucleosomes, is clear from our experiments. A related observation was reported by Allan and co-workers [62]. At a stoichiometry of one H1 molecule bound per nucleosome the affinity of each subtype for chromatin roughly correlates with the length of their C-terminal domains. The role of the C-terminal domain in specifying chromatin affinity suggests that its post-translational modifications or its interaction with other factors will have an effect on the stability of chromatin bound H1. At least, phosphorylation and acetylation of this domain have been reported [29].

The shorter NRL observed in chromatin assembled with GH1 or N-GH1 as compared to chromatin assembled with wild-type H1 or in the absence of any linker histone deserves further discussion. Association between H1 globular domains could explain this observation. The self-association of linker histones H1 and H5 has been widely reported [63]–[66] and both can be extensively cross-linked within the chromatin fibre *in situ* [67]. In the case of H5 the cross-linked region has been mapped to the globular domain [63] that interacts with supercoiled DNA in clusters of three histone molecules. Thus, the association of GH5 with DNA does not preclude the interaction with other globular domains and may even facilitate it, leading to chromatin fibre compaction. Self-association between GH1.4 could explain the shortening in spacing observed in MNase experiments when H1.4 lacking the C-terminus was added (Figure 4B). In contrast, the globular domain of H1.0 increases NRL (Figure 4C). A recent study suggests that the globular domains of individual H1 subtypes might have distinct binding geometries within the nucleosome in unperturbed chromatin [68]. In particular three charged residues in GH1.0 that interact with DNA at the nucleosomal dyad axis are replaced by neutral residues in the other somatic H1 subtypes. Additionally, when the conserved amino acids present in the other subtypes are introduced into H1.0 they compromise its capacity to bind nucleosomes. Differences in the globular domains of mouse H1.0 and H1.2 are responsible for their effect on gene expression in mouse fibroblasts [69]. Overexpression of H1.0 leads to repression while overexpression of H1.2 leads to enhanced transcription. Therefore, it seems that the globular domain of H1.0 is unique in its binding to nucleosomal DNA and this may explain its differential behaviour in the nucleosome spacing assay.

### H1 subtypes and chromatin compaction

The contribution of H1 to chromatin condensation has been a matter of debate since early studies proposed that it directs higher order chromatin folding [3], [70]. Recent studies suggested that H1 is not essential for the formation of the 30 nm fibre [71], [72]. However, inorganic cations and linker histones are clearly required to achieve full stability of condensed nucleosomal arrays [73]. Furthermore, H1 has been shown to be essential for mitotic chromosome architecture and segregation in *Xenopus laevis* egg extracts [32]. A series of studies from Rhodes group [74], [75] demonstrated that the type of folding and the degree of chromatin condensation observed changes with the addition of linker histones to the chromatin fibre. For these authors, an interdigitated solenoid fibre is only possible with chromatin containing H1, though they did not explore the role of the different H1 subtypes.

The electrostatic repulsion of the linker DNA backbone maintains the chromatin fibre in an unfolded state. The neutralization of this negative charge by binding of the H1 C-terminus or the H3 tail to DNA [76] could favour nucleosome-nucleosome interactions leading to a higher state of chromatin folding if the neutralization is produced all over the chromatin fibre. Using AFM we show that histone H1 does stabilize higher order chromatin folding, but the extent to which it does depends on the subtype. While H1.0, H1.4, H1.5 and H1x stabilize chromatin compaction, H1.1, H1.2 and to a lesser degree H1.3 maintain a relaxed chromatin structure.

The structure of compacted minichromosomes has been previously studied using cryo-electron microscopy and SV40 minichromosomes [49]. These particles contain 20–25 nucleosomes and exhibit a diverse range of compacted structures. This and previous studies [77], [78] show that SV40 minichromosomes can condense into higher order structures, such as 40 nm globules. We found that these globules were formed when minichromosomes contained the H1 subtypes that condense chromatin (Figure 2A and Figure 3). Similar structures were described when AFM was used to study cation induced condensation of chromatin fragments [79].

In general, we found a correlation between H1 subtypes affinity for chromatin and their compacting properties, but there are some exceptions. The most striking was the ubiquitously expressed H1x, which has the shortest C-terminal tail and the lowest chromatin affinity of all the somatic subtypes, yet it generates highly condensed chromatin. Interestingly, during interphase H1x is localized in condensed nucleolar chromatin [80] yet it has a high turnover rate [55].

Another unusual behaviour was found with H1.2, which has slightly higher affinity for chromatin than H1.0 but decondenses instead of compacting chromatin, as H1.0 does. Related to this, a reduction in the affinity of linker histones for chromatin has been reported [81] during the terminal differentiation of frog erythrocytes *in vivo*, when H1.0 accumulates in highly compacted chromatin. This study concluded that the affinity of linker histones for chromatin *in situ* was unrelated or inversely related to chromatin condensation. While this may be the case for H1.2, we cannot extend this conclusion to the other H1 subtypes. Intriguingly, H1.5, the subtype with the highest chromatin affinity and a strong nucleosome condenser, has been detected on active chromatin [82]–[85]. This finding could be associated with the selective removal of the other H1 subtypes or with the longer residence time of H1.5 [18], [86].

Our classification of the H1 subtypes based on their chromatin condensation properties agrees with several previous observations. For instance, H1.0 and H1x are enriched in the micrococcal nuclease-resistant part of chromatin [13], [87]. Conversely, H1.2 is enriched in soluble chromatin fractions [87], [88]. Lennox and Cohen [11] found a predominance of H1.1 and H1.2 in mouse prepachytene spermatocytes, an environment presumed to require a more open chromatin structure for genetic recombination. Khadake and Rao [23] showed by Circular Dicroism that the predominant subtypes in mammalian pachytene spermatocytes, H1.1 and H1t, were weaker condensers than the other subtypes. Moreover, H1.1 is enriched in lymphocytes, where somatic recombination is required for antibody generation [89].

The C-terminal tail of H1 occupies half of the protein length and is mainly responsible for the chromatin condensation properties, as demonstrated with the chimera H1.4–2, in which the C-terminal tail of H1.4 has been replaced with that of H1.2 (Figure 2). The chimera exhibits the chromatin decondensing properties of the H1 subtype contributing the C-terminal tail, namely H1.2.

### H1 subtypes and chromatin remodeling

ATP-dependent chromatin remodeling enzyme complexes participate in virtually every process that requires access to DNA in chromatin. The mechanism of action of these enzymatic machines has not yet been fully elucidated, but they are known to catalyze sliding of histone octamers along the DNA [90], weakening of the interaction between core histones and DNA, and H2A/H2B displacement [91], [92].

Initial experiments with nucleosome arrays suggested that the incorporation of linker histones hindered ATP-dependent remodeling [38]. We wanted to investigate possible differences between the H1 subtypes, at physiological stoichiometry, in their ability to inhibit ATP-dependent remodeling of minichromosomes. To our surprise we found that none of the somatic H1 subtypes tested had a significant effect on the action of either yeast SWI/SNF or *Drosophila* NURF, two well-known remodeling complexes that can act via different molecular mechanisms [93]. Thus, the potential barrier created by chromatin compaction in the presence of histone H1 does not inhibit chromatin remodeling of minichromosomes at the tested sites.

Though this finding does not exclude the possibility that the H1 subtypes could inhibit the accessibility to nucleosomes on different DNA sequences, it does not support the preconception that the mere incorporation of any H1 subtype abolishes chromatin remodeling of complex templates. A similar finding was reported with minichromosomes in the context of preblastodermic *Drosophila* embryo extract complemented with histone H1 [40], though the ATP-dependent remodeling complex responsible was not identified. It was later shown that the ACF complex, an ATP dependent ISWI containing complex, can remodel long nucleosomal arrays containing *Drosophila* H1 [41].

Our findings are consistent with the role played by the ATP-dependent remodeling complexes in mature chromatin assembly. The transition from a relaxed, irregularly spaced and H1 depleted structure to a fully H1 loaded, properly spaced and compacted fibre requires catalysis by chromatin remodeling complexes. ACF together with a histone chaperone (NAP-1), was shown to be sufficient for the assembly of regularly spaced nucleosomal arrays containing H1 [94]. The expression of a dominant negative form of ISWI induced chromatin decondensation of both mitotic and polythene chromosomes in *Drosophila*. These effects have been attributed to an inactive NURF complex [95], which promoted the depletion of H1 from chromosomes and subsequent chromatin decondensation. It has been reported that the knockdown of BAF53 (a key component of SWI/SNF) results in chromatin unfolding and the expansion of chromosome territories without the loss of H1 [96]. This was unexpected, as core histones and H1 are sufficient to induce higher order folding *in vitro* [75]. Along with our results these findings suggest that both NURF and SWI/SNF by catalyzing nucleosome dynamics can rearrange H1 containing chromatin thereby promoting proper nucleosome spacing and subsequently higher order folding. Nucleosome mobilization is also required and catalyzed by these remodeling complexes in the context of transcriptional activation. Thus, following the nomenclature suggested by Maier *et al.* [97] and given their ability to remodel chromatin containing histone H1, SWI/SNF and NURF are ‘chromatin remodelers’ not just ‘nucleosome remodelers’.

Our results contribute to the changing view regarding the role of the linker histones in chromatin structure and dynamics. Although H1 subtypes stabilize higher order folding of the chromatin fibre, they do it in varied ways depending on their globular and variable C-terminal domains. In all cases the resulting structure, including the 30 nm fibre, is dynamic and compatible with nucleosome movements and rearrangements promoted by suitable ATP-dependent chromatin remodelers. How exactly the various H1 subtypes influence the path of DNA between nucleosomes is an important open question, as it may explain their different effects on chromatin condensation and role.

## Materials and Methods

### Histone H1 subtype synthesis and purification

The recombinant expression in yeast and purification of human H1.0, H1.1, H1.2, H1.3, H1.4 and H1.5 was done as described by Albig *et al.* [47]. Subtype H1x was expressed in *E. coli* and purified as described in Happel *et al.* [13]. H1.0, H1.2, H1.3 and H1.5 expressed in *E. coli* are commercially available (Alexis Biochemicals). H1 protein concentration was determined with the Micro BCA Assay Kit (Pierce).

### Synthesis and purification of chimera protein H1.4–2 and H1 domain mutants

The FLAG-tagged H1.4 used in the chromatin assays was bacterially expressed from the prokaryotic expression vector pET3dH1.4/FLAG and then purified using an anti-FLAG affinity column as described previously [98]. The domain mutants of H1.4/FLAG were created using standard methods with pET3dH1.4/FLAG as the starting template. For the H1.4/H1.2/FLAG chimera, the C-terminal tail of H1.4 (residues 107–219) was replaced with the C-terminal tail of H1.2 (residues 107–213) in the pET3dH1.4/FLAG vector (Figure S4). GH1.0 was purified as described previously [63].

### Preblastodermic *Drosophila melanogaster* embryo extracts (DREX) preparation and minichromosomes assembly

The extracts were made from preblastodermic *Drosophila* embryos as described previously [99]. Minichromosomes were assembled on a 5.12 kb supercoiled plasmid containing the MMTV promoter [100] using DREX and the corresponding H1 subtype. The method was adapted from Bonte and Becker [99] and Koop *et al.* [101]. The following 50 µl reaction was incubated at 26°C for 5 hours: 11.5 µl of DREX, 38 µl of buffer A (10 mM HEPES 7.6, 100 mM KCl, 1.5 mM MgCl_2_, 0.5 mM EGTA pH 8, 10 mM β-glicerophosphate and 10 mM glycerol), 5 µl of buffer B (30 mM MgCl_2_, 2 mM DTT, 30 mM ATP, 300 mM Creatin Phosphate and 0.02 µg/µl Creatin Kinase), 212 ng plasmid DNA and between 25 and 300 ng of histone H1.

### Micrococcal nuclease (MNase) assays

The reaction was incubated for 1 minute after the addition of 1 part of MNase mix for 2 parts of assembly reaction. The MNase mix contains 0.008 U/µl of MNase (Sigma) and 6 mM CaCl_2_ in Buffer A without KCl. The reaction was stopped with 1 part of STOP mix (100 mM EDTA and 2.8% Sarcosyl), and treated first with RNase and then with Proteinase K. The purified DNA was electrophoresed through a 20 cm 1.3% agarose gel and visualized with ethidium bromide. The NRL was calculated comparing the DNA size markers with the peaks of the nucleosome ladder as described [43].

### Minichromosomes purification by MgCl_2_ precipitation

This method was first used to test the solubility of the minichromosomes assembled with each H1 subtype at increasing MgCl_2_ concentrations. Minichromosomes were incubated for 5 minutes with 5, 7.5, 10, 12.5, 15, 17.5 or 20 mM MgCl_2_ in assembly buffer containing 100 mM KCl as a monovalent cation, and then pelleted at 16,000 g for 2 minutes. Pellets and supernatants were analysed for the presence of DNA. To determine H1 stoichiometry, the same procedure was followed with the exception that the amount of minichromosomes corresponding to 12 µg of DNA was exposed to 20 mM MgCl_2_. After precipitation, the pellets were resuspended in 0.4 N H_2_SO_4_ and the soluble proteins were precipitated over night at 4°C with cold 5% TCA. The proteins were electrophoresed in a precast 16% PA gel (Invitrogen) and stained with Coomassie G-250. The bands corresponding to each linker histone subtype and histone H3 were densitometricaly measured (Quantity One software, Bio-Rad) and the ratio between them calculated.

### Minichromosome purification through sucrose gradient and sucrose cushion

To prepare the minichromosomes for AFM and the remodeling assays, we performed tandem purification through successive linear 15–30% sucrose gradient and 30% sucrose cushion ultracentrifugation. The gradients were formed in a buffer containing 100 mM KCl, 5 mM HEPES-KOH pH 7.6, 0.2 mM EDTA and Proteinase Inhibitor Cocktail (Roche). The purification proceeded as described previously [102]. The final pellet was resuspended in 100 µl gradient buffer without sucrose containing KCl at the desired concentration (20 mM for the AFM studies and 60 mM for the remodeling assays).

### Atomic Force Microscopy

We used a method adapted from [48]. Mica surface was exposed to the APTES vapours for at least 2 minutes, before deposition of 1 mM glutaraldehyde solution for 10 minutes. After successive washings with ultrafiltered (0.02 µm) water, the surface was dried with pressurised Ar. The chromatin sample was deposited at the convenient dilution over the surface and incubated for 30 min. No further dilution was needed for AFM, when the chromatin reconstitution was performed with 1 µg of DNA, and the minichromosomes then purified through the sucrose gradient and cushion and resuspended in 100 µl of buffer. Imaging was carried out with a Nanoscope III AFM (Digital Instruments, Santa Barbara, CA) using NCH silicon cantilevers (Nanosensors, Wetzlar, Germany) with a spring constant of 42 N/m. Drive amplitude was ∼20 nm with a 30% reduction set-point.

Images of two independent experiments (three for H1.3) were quantified in that way. On average 280 minichromosomes were measured for each subtype.

### Chromatin remodeling complexes purification

SWI/SNF was purified from a yeast strain provided by Dr. Craig Peterson, containing a TAP tagged SNF2 subunit as described [53]. All four Baculovirus expression vectors containing the subunits of NURF complex were kindly provided by Dr. Carl Wu. NURF complex was purified as described previously [54].

### Chromatin remodeling assays

10 ng of purified minichromosomes were dissolved in a buffer containing 10 mM HEPES-KOH pH 7.6, 60 mM KCl, 5 mM MgCl_2_, 1 mM ATP and 0.7 mM DTT. They were incubated for 30 minutes at 30°C in the presence of 12 U of *Fok* I restriction enzyme and increasing concentrations of either ySWI/SNF or dNURF complexes. The reactions were stopped with buffer containing 100 mM EDTA and 2.8% Sarcosyl and subsequently deproteinized. The resulting DNA was cut with 1 U of *Hinf* I restriction enzyme, purified again and used as template for 20 cycles of linear PCR extension with radiolabeled oligonucleotides that hybridize to sequences in the nucleosome neighbouring the *Fok* I cleavage site (Figure S5). After purifying the linear PCR products, the samples where electrophoresed through a denaturing 10% polyacrylamide gel.

### Mononucleosome assembly and H1 binding

A 220 bp DNA fragment corresponding to Nuc B of MMTV promoter was amplified and used for mononucleosome assembly. Nucleosomes were reconstituted by the salt dialysis method [103] using HeLa core histones. To test H1 subtypes binding, increasing amounts of H1 were incubated with 30 ng of preformed mononucleosomes in a buffer containing 10 mM Tris-HCl, 50 mM KCl and 6% glycerol. After 20–25 min at RT, the reactions were loaded on a 0.7% agarose gel to test for incorporation. To analyse binding of the H1 domain mutants to mononucleosomes, the same procedure was followed, with the exception that the DNA fragment was radiolabeled and purified through a 10–30% glycerol gradient.

## Supporting Information

Figure S1 Effect of MgCl2 concentration on chromatin solubility. Minichromosomes assembled with each H1 subtype yielding a NRL of 200 bp were incubated for 5 minutes with increasing concentrations of MgCl2 (5, 7.5, 10, 12.5, 15, 17.5 and 20 mM) in assembly buffer containing 100 mM KCl as a monovalent cation, and the insoluble chromatin was sedimented at 16,000×g. The pellet (P) and the supernatant (SN) were deproteinized and analyzed on a 0.7% agarose gel (upper panel). Bands corresponding to both fractions were quantified (Quantity One, Bio-Rad) and the result represented as the percentage of the soluble chromatin at each MgCl2 concentration (lower panel). We used this methodology for purifying minichromosomes and determining histone H1 stoichiometry (Figure 1C). 50% of precipitation was reached at 15 mM MgCl2 for minichromosomes without H1. Minichromosomes containing the H1 subtypes were precipitated at lower MgCl2 concentration. Precipitation of 50% was reached between 10 and 12.5 mM MgCl2, with the exception of minichromosomes containing H1x, which achieved complete precipitation at 10 mM MgCl2. Those containing H1.1 behaved like minichromosomes without H1. At 20 mM MgCl2 all minichromosomes were precipitated no matter the H1 subtype added. Therefore we chose this concentration of MgCl2 in our studies of H1 stoichiometry (Figure 1C and E).
(0.08 MB PDF)

Figure S2 Histone H1.4 binding to mononucleosomes. Centrally positioned mononucleosomes were assembled with a 220 bp DNA fragment corresponding to the nucleosome B sequence in the MMTV promoter. After the purification step, they were incubated with increasing amounts of H1.4 and analysed on a 0.7% (w/v) agarose gel. The amount of the protein is indicated.
(0.07 MB PDF)

Figure S3 H1.4 domains contribution to chromatin compaction. 2 µm AFM images of minichromosomes assembled without H1 or with H1.4 domain mutants. GH1.4, corresponds to the globular domain (GD), N-GH1.4 corresponds to GD with the N terminus and GH1.4-C is the GD plus the C-terminal domain. The horizontal scale bar corresponds to 200 nm. The vertical scale in nm is shown.
(0.39 MB PDF)

Figure S4 Multiple alignment of H1 subtypes including H1t. The H1.4 C-terminal domain is underlined. This is the sequence of the protein that was substituted by the equivalent part of the H1.2 subtype to built the H1.4-2 chimera protein.
(0.03 MB PDF)

Figure S5 Schematic representation of the strategy used to measure chromatin remodeling. During chromatin remodeling process Fok I restriction enzyme gain access to its target sites. After the reaction is stopped, DNA is then cut with Hinf I restriction enzyme. Finally, a primer extension with a labelled primer (either primer 1 or 2) was performed from indicated sites, generating two types of fragments per primer, depending on the previous accessibility of Fok I. Primer 1 was used to monitor accessibility in nucleosome B and primer 2 was used to monitor accessibility in nucleosome A.
(0.01 MB PDF)
