# Supplementary figures and images for: Histone H1 Subtypes Differentially Modulate Chromatin Condensation without Preventing ATP-Dependent Remodeling by SWI/SNF or NURF
Source: PLoS One. 2009 Oct 1;4(10):e0007243. doi: 10.1371/journal.pone.0007243 (PMC2748705; doi:10.1371/journal.pone.0007243)

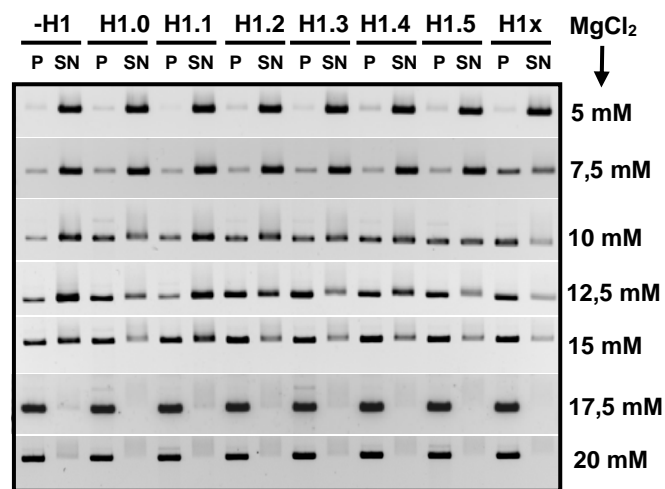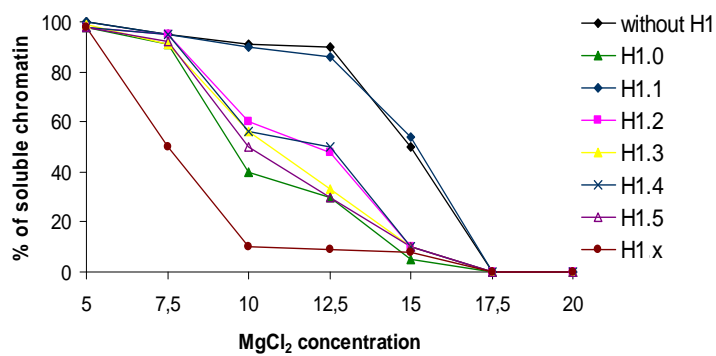

**Figure S1**

Supplement: Figure S1 — Effect of MgCl2 concentration on chromatin solubility. Minichromosomes assembled with each H1 subtype yielding a NRL of 200 bp were incubated for 5 minutes with increasing concentrations of MgCl2 (5, 7.5, 10, 12.5, 15, 17.5 and 20 mM) in assembly buffer containing 100 mM KCl as a monovalent cation, and the insoluble chromatin was sedimented at 16,000×g. The pellet (P) and the supernatant (SN) were deproteinized and analyzed on a 0.7% agarose gel (upper panel). Bands corresponding to both fractions were quantified (Quantity One, Bio-Rad) and the result represented as the percentage of the soluble chromatin at each MgCl2 concentration (lower panel). We used this methodology for purifying minichromosomes and determining histone H1 stoichiometry (Figure 1C). 50% of precipitation was reached at 15 mM MgCl2 for minichromosomes without H1. Minichromosomes containing the H1 subtypes were precipitated at lower MgCl2 concentration. Precipitation of 50% was reached between 10 and 12.5 mM MgCl2, with the exception of minichromosomes containing H1x, which achieved complete precipitation at 10 mM MgCl2. Those containing H1.1 behaved like minichromosomes without H1. At 20 mM MgCl2 all minichromosomes were precipitated no matter the H1 subtype added. Therefore we chose this concentration of MgCl2 in our studies of H1 stoichiometry (Figure 1C and E). (0.08 MB PDF) [file pone.0007243.s001.pdf]

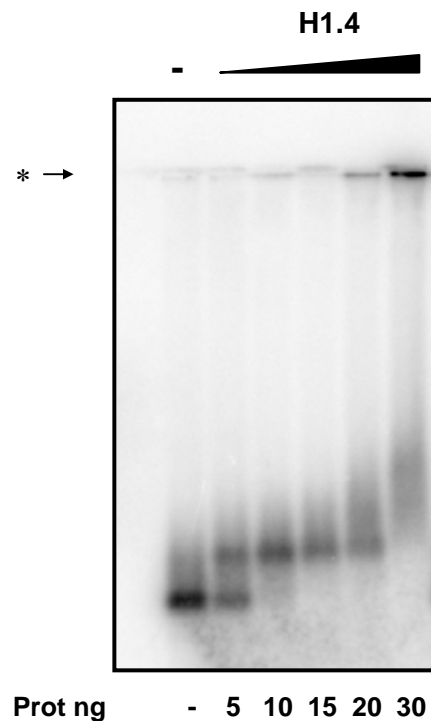

**Figure S2**

Supplement: Figure S2 — Histone H1.4 binding to mononucleosomes. Centrally positioned mononucleosomes were assembled with a 220 bp DNA fragment corresponding to the nucleosome B sequence in the MMTV promoter. After the purification step, they were incubated with increasing amounts of H1.4 and analysed on a 0.7% (w/v) agarose gel. The amount of the protein is indicated. (0.07 MB PDF) [file pone.0007243.s002.pdf]

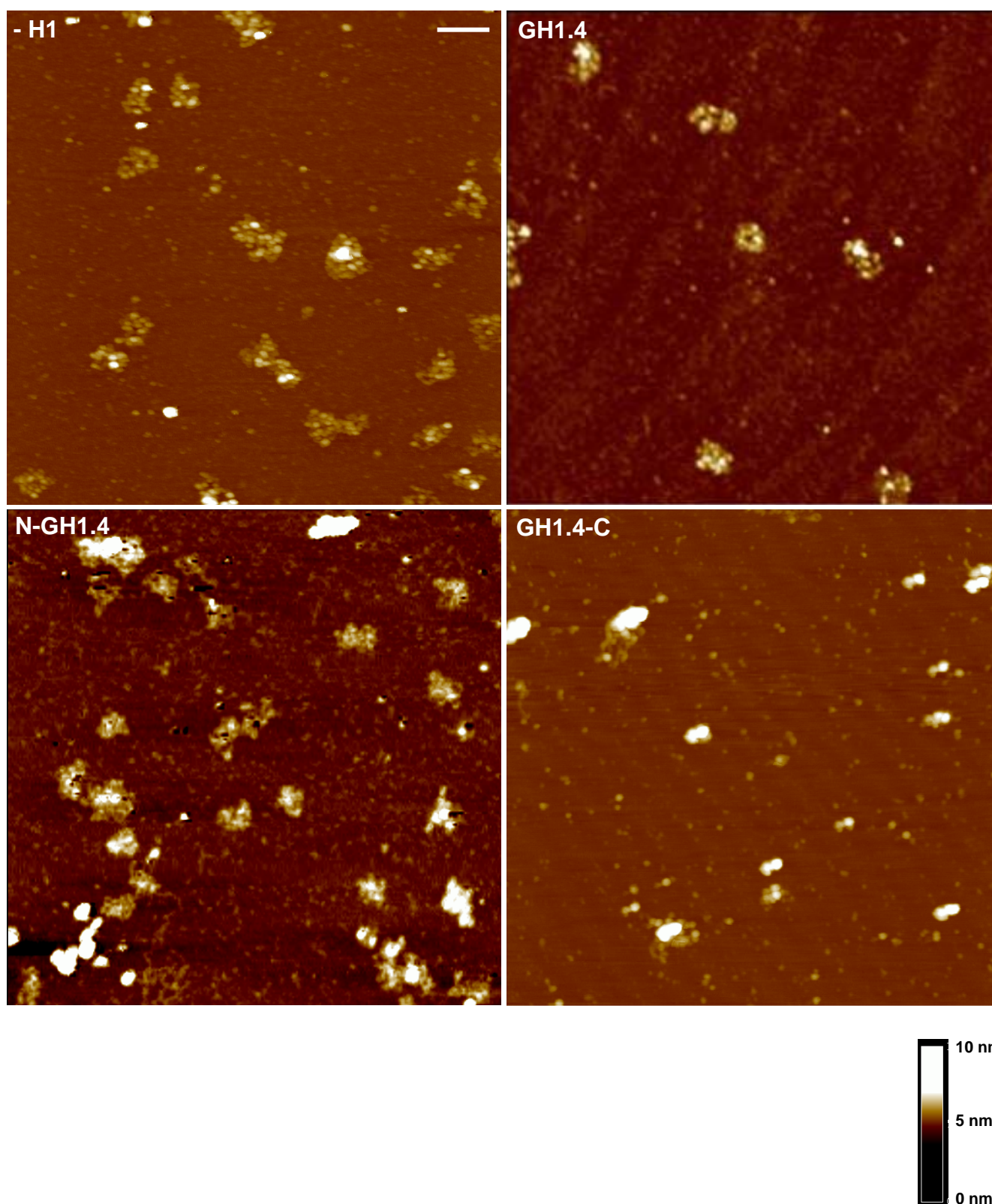

**Figure S3**

Supplement: Figure S3 — H1.4 domains contribution to chromatin compaction. 2 µm AFM images of minichromosomes assembled without H1 or with H1.4 domain mutants. GH1.4, corresponds to the globular domain (GD), N-GH1.4 corresponds to GD with the N terminus and GH1.4-C is the GD plus the C-terminal domain. The horizontal scale bar corresponds to 200 nm. The vertical scale in nm is shown. (0.39 MB PDF) [file pone.0007243.s003.pdf]

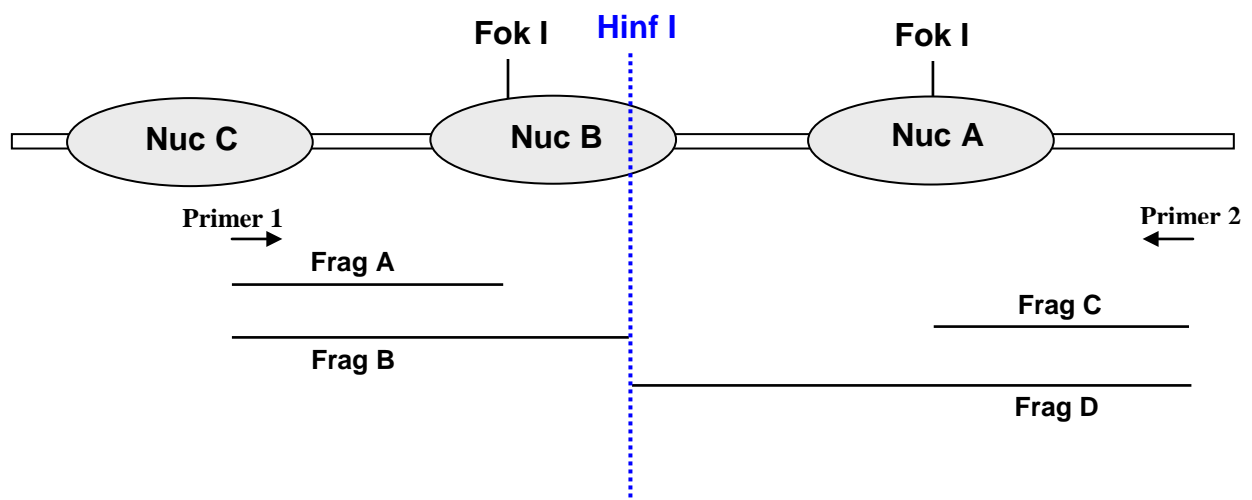

**Figure S5**

Supplement: Figure S5 — Schematic representation of the strategy used to measure chromatin remodeling. During chromatin remodeling process Fok I restriction enzyme gain access to its target sites. After the reaction is stopped, DNA is then cut with Hinf I restriction enzyme. Finally, a primer extension with a labelled primer (either primer 1 or 2) was performed from indicated sites, generating two types of fragments per primer, depending on the previous accessibility of Fok I. Primer 1 was used to monitor accessibility in nucleosome B and primer 2 was used to monitor accessibility in nucleosome A. (0.01 MB PDF) [file pone.0007243.s005.pdf]
