# Supplementary material for: Histone H1 Subtypes Differentially Modulate Chromatin Condensation without Preventing ATP-Dependent Remodeling by SWI/SNF or NURF
Source: PLoS One. 2009 Oct 1;4(10):e0007243. doi: 10.1371/journal.pone.0007243 (PMC2748705; doi:10.1371/journal.pone.0007243)
Supplement: Figure S4 — Multiple alignment of H1 subtypes including H1t. The H1.4 C-terminal domain is underlined. This is the sequence of the protein that was substituted by the equivalent part of the H1.2 subtype to built the H1.4-2 chimera protein. (0.03 MB PDF) [file pone.0007243.s004.pdf]

```
H1.5    -MSETAPAETATP--APVEKSPAKKKATKKAAGAGAAKRKATGPPVSELITKAVAASERNGLSLA-ALKKALAAGGYDVEKNNSRIKLGLKSLVSKGTLVQTKGTGASGSFKLNKK
H1.4    -MSETAPAAPAAP--APAEKTPVKKKARKS---AGAAKRKASGPPVSELITKAVAASERSGVSLA-ALKKALAAAGYDVEKNNSRIKLGLKSLVSKGTLVQTKGTGASGSFKLNKK
H1.2    -MSETAPAAPAAA--PPAEKAPVKKKAAKKAG-GTP--RKASGPPVSELITKAVAASERSGVSLA-ALKKALAAAGYDVEKNNSRIKLGLKSLVSKGTLVQTKGTGASGSFKLNKK
H1.3    -MSETAPLAPTIP--APAEKTPVKKK-AKKAG-ATAGKRKASGPPVSELITKAVAASERSGVSLA-ALKKALAAAGYDVEKNNSRIKLGLKSLVSKGTLVQTKGTGASGSFKLNKK
H1.1    -MSETVPPAPAAS--AAPEKPLAGKKAKKPAKAAAASKKKPAGPSVSELIVQAASSSERGGVSLA-ALKKALAAAGYDVEKNNSRIKLGIKSLVSKGTLVQTKGTGASGSFKLNKK
H1.T    -MSETVPAASASAGVAAMEKLPTKKRGRKPAG-LISASRKVPNLSVSKLITEALSVSERVGMSLV-ALKKALAAAGYDVEKNNSRIKLSLKSLVNKGILVQTRGTGASGSFKLSKK
H1.0    -MTENSTSAPAAK--------PKRAKASKKST---------DHPKYSDMIVAAIQAENRAGSSRQ-SIQKYIKSHYKVGENAHSQIKLSIKRLVTTGVLKQTKGVGASGSFRLAKS
H1.X    MSVELEEALPVTTAEG-MAKKVTKAGGSAALSPSKKRKNSKKKNQPGKYSQLVVETIRRLERNGSSLAKIYTEAKKVPWFDQQNGRTYLKYSIKALVQNDTLLQVKGTGANGSFKLNRK

H1.5    AASGEAKPKAKKAGAAKAKKPAGAT--PKKAKKAAGAKKAVKKTPKKAKKPAAAGV-KKV
H1.4    AASGEAKPKAKKAGAAKAKKPAGAAKKPKKATGAATPKKSAKKTPKKAKKPAAAAG-AKK
H1.2    AASGEAKPKVKKAGGTKPKKPVGAAKKPKKAAGGATPKKSAKKTPKKAKKPAAATVTKKV
H1.3    AASGEGKPKAKKAGAAKPRKPAGAAKKPKKVAGAATPKKSIKKTPKKVKKPATAAGTKKV
H1.1    ASSVETKPGASKV--ATKTKATGASKKLKKATGAS--KKSVK-TPKKAKKPAATRK---S
H1.T    VIPKSTRSKAKKSVSAKTKK--------LVLSRDSKSPKTAK-TNKRAKKPRATTP--KT
H1.0    DEPKKSVAFKKTKKEIKKVATPKKASKPKKAASKAPTKKPKATPVKKAKKKLAATP--KK
H1.X    KLEGGGE---RRGAPAAATAPAPTAHKAKKAAPGAAGSRRADKKPARGQKPEQRSH--KK
```

Figure S4

Clausell et al.
